# Bioinspired catecholamine-derived AuNPs@polynorepinephrine@MWCNT electroactive nanomaterial for real-time monitoring of glucose dynamics

**DOI:** 10.1007/s00604-026-08201-5

**Published:** 2026-06-17

**Authors:** Artur Jędrzak, Maria Kuznowicz, Teofil Jesionowski

**Affiliations:** 1 Institute of Chemical Technology and Engineering, Faculty of Chemical Technology, Poznan University of Technology, Berdychowo 4, Poznan, PL-61131 Poland; 2 Interdisciplinary Centre for Ecotechnology, Poznan University of Technology, Poznan, PL-60965 Poland

**Keywords:** Wireless sensor, Screen-printed electrode, Multi-walled carbon nanotubes, Chronoamperometry, Point-of-care testing, Polynorepinephrine, Catecholamine, Glucose dynamics

## Abstract

A bioinspired nanomaterial is introduced based on polynorepinephrine (PNE) grafted to multi-walled carbon nanotubes (MWCNT) with gold nanoparticle decoration (Au NPs), developed as an advanced electroactive interface for glucose biosensing. The nanoplatform was deposited on a screen-printed electrode (SPE) with glucose oxidase (GOx) from *Aspergillus niger* for potential use in point-of-care testing based on a rapid electrocatalytic response to glucose. This molecular-level modification strategy provides an effective approach to tailor catecholamine-derived polymers and improve their electrochemical performance. This biosensor demonstrated a wide linear range (0.1–30.0 mM), with a limit of detection of 2.15 µM and a limit of quantification of 6.52 µM, and sustained stability up to 20 weeks. The biosensor was used to perform in vitro tests with other analytical devices, including a clinical glucometer and a home glucometer. The repeatability and reproducibility of the biosensor were also tested, as well as the possibility of collection of data on changing levels of blood glucose throughout the day. The results demonstrate that the proposed polynorepinephrine-based biosensor represents a promising alternative electroactive coating to the widely studied polydopamine. From an engineering standpoint, the synthesis is compatible with fabrication of screen-printed electrodes. This platform underscores its suitability for potential decentralized point-of-care testing.

**Graphical Abstract:**

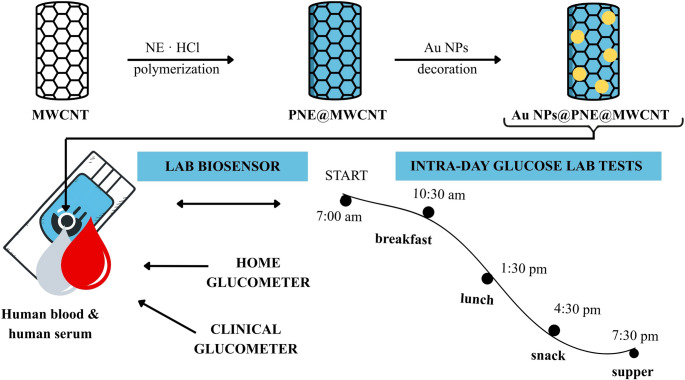

**Supplementary Information:**

The online version contains supplementary material available at 10.1007/s00604-026-08201-5.

## Introduction

As reported by the International Diabetes Federation (IDF), in 2021, around 537 million adults aged 20 to 79 worldwide lived with diabetes. This represents about 10.5% of the population within that age range. The diabetic population is expected to increase to 643 million by 2030 and to 783 million by 2045, with a more significant increase expected in low- and middle-income nations [1].

Reliable, accurate, and fast-response glucose biosensors still face significant challenges related to the 3 S principle, which requires improving sensitivity, increasing selectivity, and ensuring long-term stability. Additionally, it is necessary to solve problems related to interferences caused by substances present in body fluids, including salt ions influencing enzyme activity [2], to enable wireless data transmission and archiving [3], and finally, to attempt to reduce the cost of components [4].

One of the promising solutions to these challenges arises from the growing research on the participation of nanomaterials in improving the performance of biosensors, increasing their sensitivity, stability, and signal amplification [5]. Nature-inspired nanomaterials also provide increased interaction between the biomolecule and the target analyte due to their high surface area-to-volume ratio, resulting in more sensitive detection [6].

Biomimetic nanomaterials include catecholamine-based coatings inspired by mussel adhesive chemistry, which are widely recognized for their strong adhesion, surface versatility, and biocompatibility. Among them, polydopamine (PDA) is one of the most extensively studied systems, offering uniform surface coverage, protection against oxidation, and facile biofunctionalization due to the presence of multiple reactive functional groups [7, 8]. In this context, polynorepinephrine (PNE), a structurally related but less explored analogue, has recently emerged as a promising alternative. Compared to PDA, the self-polymerization of PNE precursors proceeds more slowly, which can result in coatings with lower surface roughness, improved uniformity, and potentially enhanced biocompatibility. These features may play an important role in modulating the kinetics of electrochemical processes and overall biosensor performance [9]. Unlike PDA, which may exhibit heterogeneous coating formation and uneven distribution of reactive groups leading to less controlled enzyme immobilization, polynorepinephrine provides additional β-hydroxyl and amine functionalities that promote increased hydrogen bonding and improved hydrophilicity. These features may facilitate more efficient and reproducible biomolecule immobilization, enhanced interfacial stability, and improved electron transfer, making polynorepinephrine particularly attractive for electrochemical biosensing applications [10–13]. Furthermore, in our previous work, we showed, that polynorepinephrine exhibited better enzyme loading properties, which may contribute to improved biosensor properties [14].

Metal, metal oxides, and carbon-based nanomaterials can provide better environmental stability to the sensing platform, increasing the overall stability and durability of the biosensor while also improving conductivity. Decoration with gold nanoparticles (Au NPs) increases the electrochemically active surface area, minimizes the biomolecule’s impedance [15], and improves enzyme immobilization [16]. Moreover, specific nanomaterials may serve as signal boosters, which improve the identification of weak signals, this being essential for the detection of low analyte concentrations [17]. Such materials include multi-walled carbon nanotubes (MWCNT), which significantly improve conductivity by amplifying the signal from the receptor layer [18].

Several studies have demonstrated that the combination of AuNPs and MWCNTs leads to synergistic effects, resulting in improved enzyme loading, enhanced stability, and reduced charge transfer resistance compared to single-component systems [19, 20]. Similar nanostructured electrochemical sensing strategies have also been explored in wearable and non-invasive glucose monitoring platforms [21]. For instance, AuNP-decorated carbon nanotube platforms have been shown to provide improved sensitivity and faster electron transfer kinetics in glucose biosensors, while also stabilizing the immobilized enzyme layer [22, 23]. The enhanced sensitivity observed after AuNP incorporation may result from the combined effect of reduced charge-transfer resistance and increased electroactive surface area, leading to improved electron-transfer kinetics and a higher density of electroactive sites [24, 25]. Therefore, the use of MWCNT–AuNP hybrid structures represents an effective strategy to overcome common limitations in biosensor design and to improve the overall analytical performance of enzyme-based sensing systems [26, 27].

Herein, we propose a bioinspired polynorepinephrine-based electroactive interface as an alternative to conventional catecholamine coatings, addressing the limited control over enzyme immobilization efficiency and electron-transfer kinetics observed in widely used nanomaterials, which often translates into compromised sensitivity and detection limits in glucose biosensing applications. We report here on the first comprehensive study to analyse a nanomaterial based on PNE grafted to MWCNT with Au NP decoration, used to support wireless glucose sensing in model and real samples, as a potential point-of-care testing (POCT) tool, in comparison with a commercial home glucometer and a clinical glucometer. In addition, this biosensor has been applied in testing the body’s changing blood glucose levels throughout the day.

The paper opens several avenues for the future development of even more efficient analytical biosensors for medical purposes, including the most common application of measuring glucose in diabetics.

## Experimental

### Materials and reagents

Norepinephrine hydrochloride (NE · HCl), tris(hydroxymethyl) aminomethane (TRIS) (pH 8.5, 10 mM), phosphate buffer saline (PBS) (pH 7.4, 100 mM), multi-walled carbon nanotubes (MWCNT), glucose oxidase (GOx) from *Aspergillus niger* (protein content 65–85%, molecular weight 160 kDa), α-D-Glucose (≥ 99.5%, MW: 180.16 g mol^− 1^), (hydroxymethyl)ferrocene (FcMeOH), human serum from male AB plasma, bovine serum albumin (BSA), tetrachloroauric acid (HAuCl_4_), chitosan (Chit), sodium borohydride (NaBH_4_), fructose (≥ 99.5%), uric acid (≥ 99%), ascorbic acid (≥ 99%), dopamine (≥ 98%), potassium ferrocyanide, potassium ferricyanide were purchased from Merck, Poland. Electrochemical measurements were performed using a three-electrode screen-printed sensor (ItalSens Carbon SPE, PalmSens, Houten, Netherlands) comprising a graphite working electrode (Ø 3 mm, geometric area 7.07 mm²), a carbon counter electrode, and a silver pseudo-reference electrode (Ag/AgCl). All of the chemicals were of analytical grade.

### Synthesis of Au NPs@PNE@MWCNT nanomaterial

First, 10 mg of multi-walled carbon nanotubes were ultrasonically dispersed in 20 mL of TRIS solution (10 mM, pH 8.5). To this, 10 mg of norepinephrine hydrochloride was added, and the system was stirred at ambient temperature for 24 h with access to oxygen as an oxidizing agent. After this time, the material was cleaned three times using Milli-Q^®^ water.

For the preparation of gold nanoparticles@polynorepinephrine@ multi-walled carbon nanotubes, 20 mg of PNE@MWCNT was dispersed in 10 mL of distilled water. Next, 1.0 mL of an aqueous tetrachloroauric acid solution was added and stirred for 15 min. After that, 2.5 mL of a freshly prepared aqueous sodium borohydride solution (6 mg mL^−^¹) was gradually added with continuous stirring for 4 h at ambient temperature to complete the reaction. Finally, the reaction mixture was centrifuged at 6000 rpm and washed thoroughly with water. The material was stored in solution in a refrigerator until use.

### Preparation of biosensor

**Fig. 1:**
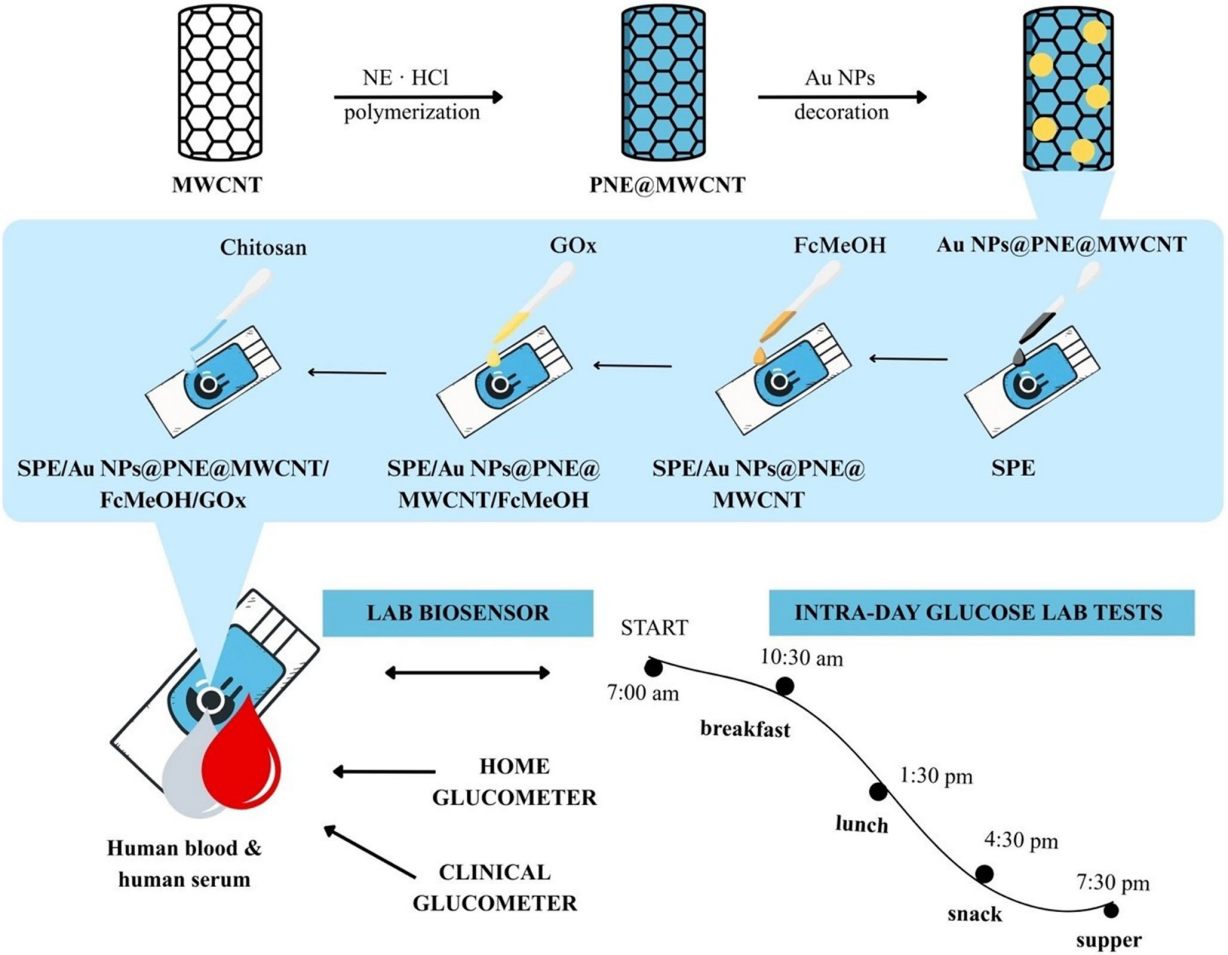
General concept of the work, including the individual stages of synthesis and electrode modification

To fabricate the biosensor, a screen-printed electrode (SPE) was used. In the first step, the electrode was functionalized with 2 µL of the hybrid Au NPs@PNE@MWCNT material and left to dry (5 mg mL⁻¹). The carbon working electrode of the SPE provides a chemically stable and conductive surface that enables strong π–π interactions with MWCNTs, ensuring good adhesion and efficient electron transfer. The deposited Au NPs@PNE@MWCNT composite forms a conductive and porous layer, where polynorepinephrine acts as an adhesive matrix, promoting strong interfacial interactions between the carbon surface, MWCNTs, and gold nanoparticles through hydrogen bonding and catechol-metal coordination. Subsequently, 2 µL of a mediator solution (5 mg mL⁻¹) was deposited onto the surface of the working electrode and dried. FcMeOH enables efficient redox mediation due to hydrophobic interactions with the nanocomposite layer, facilitating fast electron shuttling between the enzyme active sites and the electrode surface [28, 29]. Then, 2 µL of GOx was applied (5 mg mL^− 1^). Finally, the entire structure was treated with 2 µL of a 1% chitosan dissolved in acetic acid is used as a protective and stabilizing layer. Chitosan forms a uniform film which can improve mechanical stability, and preventing leaching of the active materials [30, 31]. The individual steps of electrode modification and the general concept of work is shown in Fig. 1.

### Physicochemical characterization

Transmission electron microscopy (TEM) analysis was carried out using a Jeol JEM 1400 analyser. Atomic force microscopy (AFM) was performed using an Agilent 5500 in intermittent contact mode, under atmospheric conditions. To test the stability of the materials in a liquid solvent, zeta potential (ζ) values were determined using a Zetasizer Nano ZS with a range of 0.6–6000 nm. X-ray photoelectron spectroscopy analyses (XPS) were performed in a multi-chamber ultra-high vacuum (UHV) analytical system manufactured by Prevac.

### Electrochemical measurements

All electrochemical measurements were performed using the Sensit Wearable potentiostat with the dedicated PStouch application (Palmsens, Netherlands). The potentiostat operates in a wireless mode, enabling bidirectional data communication via Bluetooth with an external mobile device. Electrochemical signals are acquired, digitized, and transmitted in real time to the PStouch software, where they are stored and processed. The compact architecture makes these potentiostats particularly suitable for point-of-care testing (POCT). Different concentrations of glucose solution were dissolved in 50 mM PBS (pH 7.4) and examined using cyclic voltammetry (CV) and chronoamperometry (CA). The potential was set between − 0.2 and 0.6 V for CV testing, and the scan rate was 10 mV s^− 1^. The constant potential for glucose detection by CA was set at 0.3 V. The sensor was stored in a refrigerator and tested every two weeks (for 20 weeks) to establish time-stability. The response of the biosensor in glucose detection was evaluated in the presence of potential interferents such as fructose, L-cysteine, uric acid, ascorbic acid, and dopamine.

Glucose measurements were performed using a Wellion^®^ electrochemical home-use glucometer for self-monitoring and an iXell^®^ clinical-grade glucometer as the reference, both employing disposable test strips and electrochemical enzymatic detection. The exact specification is provided in Table S1 (see Supplementary Material).

## Results and discussion

### Morphological characterization

**Fig. 2:**
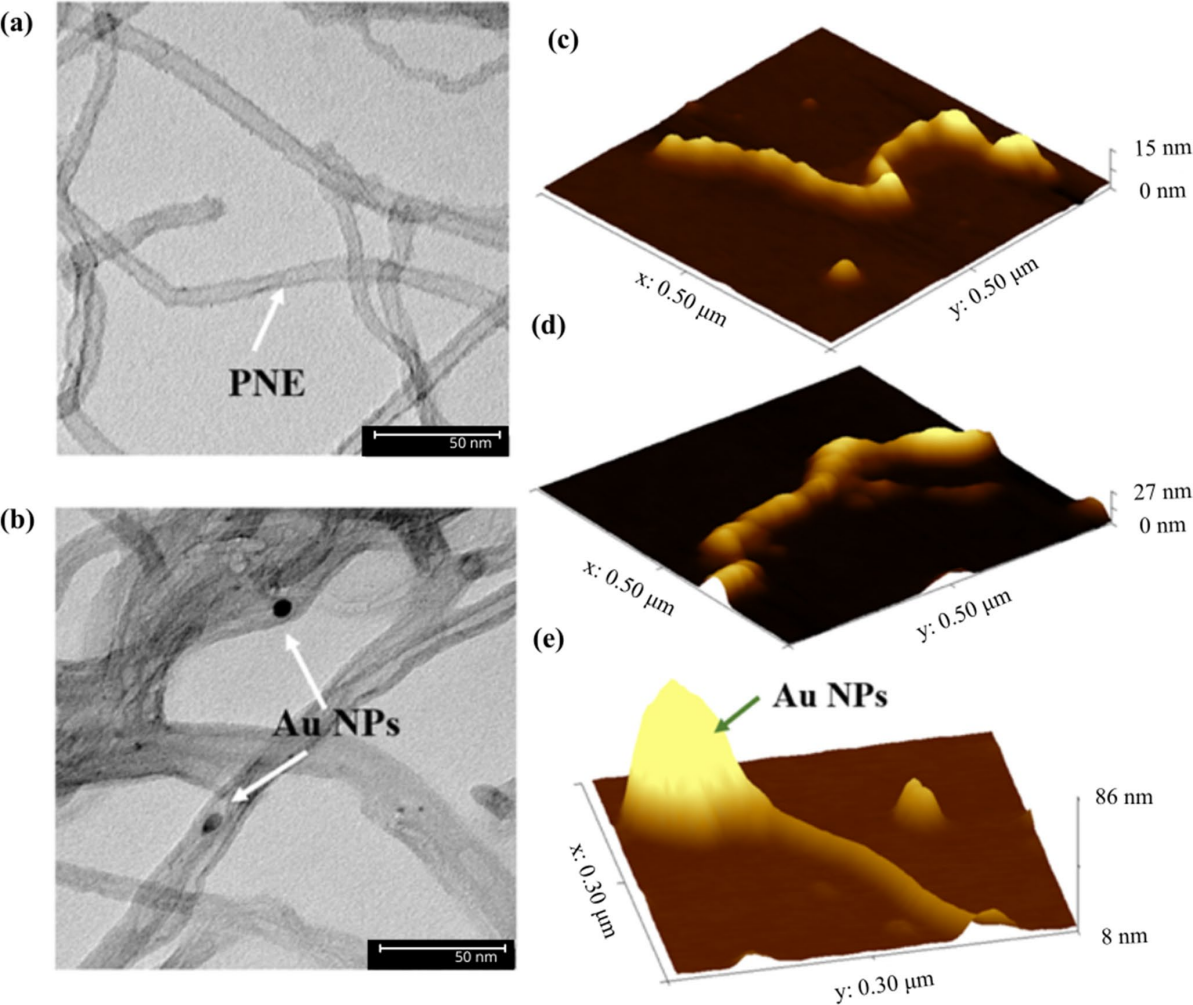
TEM micrographs of: PNE@MWCNT (**a**); Au NPs@PNE@MWCNT (**b**); AFM of: MWCNT (**c**); PNE@MWCNT (**d**); Au NPs@PNE@MWCNT (**e**)

The TEM (Fig. 2a) shows an MWCNT coated with a polynorepinephrine biopolymer. This layer appears as a heterogeneous, contrasting coating on the nanotube surface, of the order of 3–4 nm in size, indicating successful functionalization. Figure 2b shows gold nanoparticles distributed on the nanotube surface. TEM analysis findings confirm that both biopolymer functionalization and gold nanoparticle deposition are effective. Observations indicate the formation of hybrid Au NPs@PNE@MWCNT structures, which may significantly improve the electrical and catalytic properties of the material, as well as its potential for application in biosensor systems and nanocomposites. The gold nanoparticles had a diameter of 6–18 nm. Additional TEM images of Au NPs@PNE@MWCNT with distribution histogram are shown in Fig S1 (see Supplementary material).

The AFM images obtained are presented in Fig. 2, enabling a comparison of the morphologies of the different materials. In Fig. 2c, the MWCNT are characterized by a stretched structure with a regular topography, with clearly defined fibrous elements. In Fig. 2d, the nanotubes are covered with a biopolymer layer, which can be observed from the increase in the volume of the structure and a noticeable increase in the value on the z-axis, indicating the presence of an additional surface layer.

Figure 2e shows a gold nanoparticle embedded at the tip of the nanotube, where the z-axis growth is most pronounced. In addition to the terminal attachment, gold nanoparticles were also observed to preferentially anchor at structural defect sites along the nanotube walls, such as vacancies or step edges, where the local surface energy is higher. This behaviour indicates that the defect regions serve as nucleation centres, facilitating nanoparticle attachment. Such defect-assisted deposition contributes to increased surface roughness and may enhance the catalytic or electronic performance of the composite material.

The zeta potential measurements revealed significant changes in the surface charge electrokinetic potential during subsequent stages of nanomaterial modification. For unmodified multi-walled carbon nanotubes the zeta potential was − 6.24 mV, indicating moderate colloidal instability. Coating the nanotubes with a layer of polynorepinephrine (PNE@MWCNT) resulted in a significant reduction in zeta potential to -19.72 mV, suggesting an increased number of functional groups imparting a stronger negative charge to the particle and thus improving colloidal stability. Modification with gold nanoparticles (Au NPs@PNE@MWCNT), on the other hand, increased the zeta potential to -15.31 mV. The results obtained confirm the effectiveness of stepwise functionalization of MWCNT, and indicate the possibility of controlling their surface properties through appropriately designed modification processes. The increase in zeta potential after the incorporation of Au nanoparticles can be attributed to changes in the surface charge of the nanocomposite resulting from interactions between Au nanoparticles and the functional groups present in the polymer matrix. The incorporation of metallic nanoparticles may modify the surface chemistry and charge distribution of the composite, leading to a shift in the measured zeta potential [32, 33].

Consistently, the stable zeta potential correlates with the observed long-term electrochemical performance, as it reflects the stabilization provided by the Au NPs@polynorepinephrine@MWCNT composite. The polynorepinephrine matrix contributes surface functional groups that promote interfacial interactions, while MWCNTs and gold nanoparticles form a conductive and mechanically robust network. Together, these features limit aggregation, suppress material leaching, and preserve enzyme immobilization, resulting in minimal signal degradation during extended storage and repeated electrochemical measurements.

**Fig. 3:**
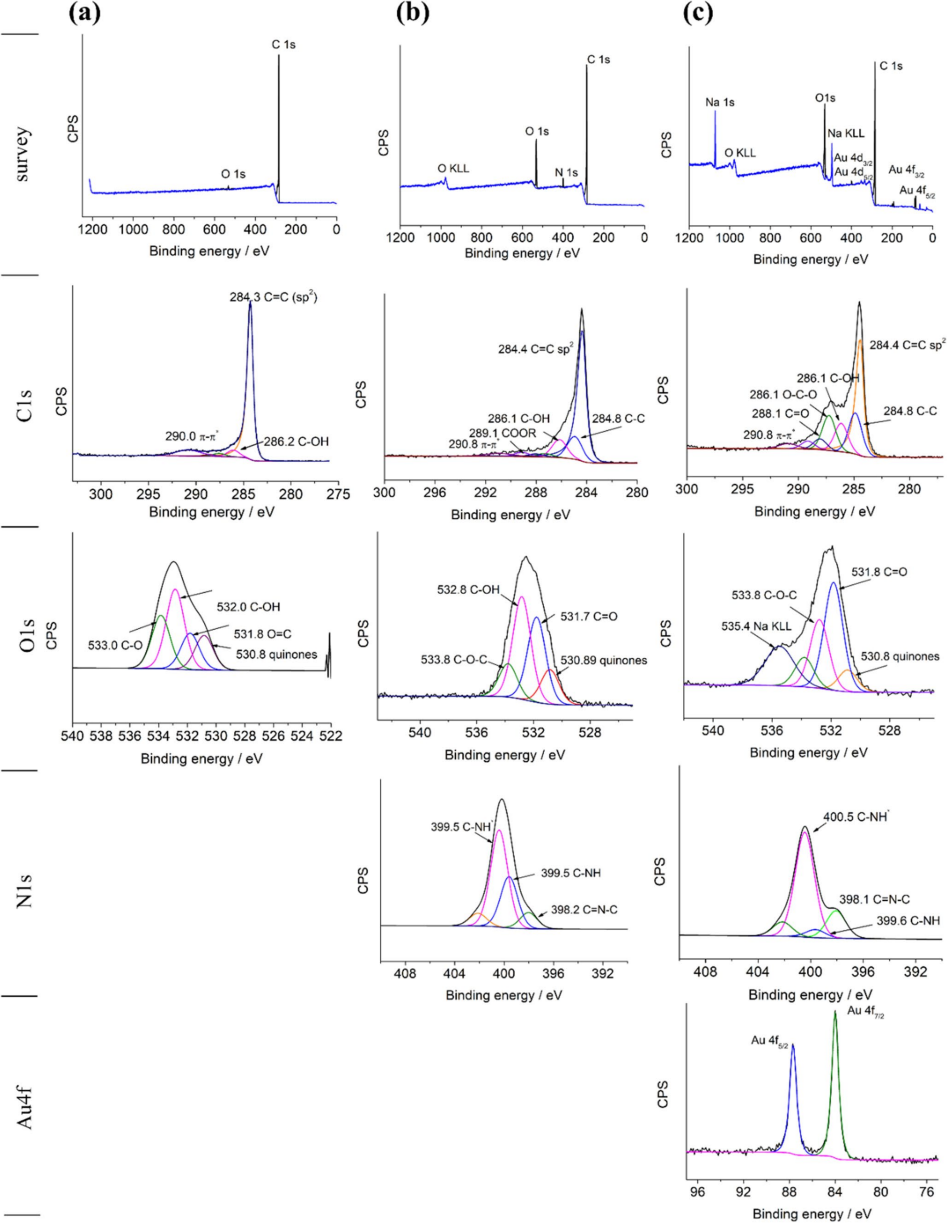
XPS spectra of: MWCNT (**a**); PNE@MWCNT (**b**); Au NPs@PNE@MWCNT (**c**)

The surface chemistry of the pristine and modified MWCNT was analysed using X-ray photoelectron spectroscopy (XPS), and the resulting spectra are shown in Fig. 3.

In the XPS spectra of the reference unmodified multi-walled carbon nanotubes (Fig. 3a), the C 1s signal dominates, with a characteristic maximum corresponding to sp² carbon in the graphitic structure (284.3 eV). A weak O 1s signal is also observed (524.4 eV), originating from natural surface passivation and oxygen-containing groups at structural defects.

After functionalization with the biopolymer (Fig. 3b), an increase in the intensity of the O 1s peak is observed (529.4 eV) together with the appearance of additional components in the C 1s region corresponding to C–O, C = O, and O–C = O bonds. The surface modification of MWCNT involving coating them with a polynorepinephrine layer resulted in the appearance of a nitrogen signal in the XPS spectra, which was absent in the case of the unmodified nanotubes. The presence of this signal, located in the region corresponding to amine groups and their protonated forms, clearly confirms the introduction of nitrogen functionalities derived from polynorepinephrine into the nanotube surface layers.

The π–π stacking interactions between the aromatic rings of the nanotubes and the catechol groups of polynorepinephrine dominate between MWCNT and polynorepinephrine. Additionally, van der Waals interactions and hydrogen bonds are present between the hydroxyl (–OH) and amine (–NH₂) groups of PNE and the carbon surface.

In samples containing gold nanoparticles (Fig. 3c), characteristic Au 4f peaks (Au 4f₇/₂ and Au 4f₅/₂) are clearly detected, with binding energy positions corresponding to metallic gold. The catechol and amine groups of PNE can act as reducing and anchoring centres, enabling the chemisorption of Au nanoparticles through coordination of gold atoms with electron donors (O, N). Both Au–O and Au–N bonds are formed, stabilizing the AuNPs on the biopolymer surface.

Quantitative XPS analysis further confirmed the stepwise surface functionalization of the composite. Pristine MWCNTs consisted predominantly of carbon (98.8 at%) with minor oxygen contributions (1.2 at%). After PNE deposition, the appearance of nitrogen (3.3 at%) and an increased oxygen content (11.5 at%) verified successful polymer coating. Following incorporation of gold nanoparticles, the Au NPs@PNE@MWCNT composite exhibited detectable Au 4f signals (0.4 at%), accompanied by nitrogen (1.0 at%) and oxygen (16.7 at%), confirming the presence of both the polymer matrix and metallic nanoparticles. High-resolution N 1s spectra were deconvoluted into components corresponding to amine/amine-like and oxidized nitrogen species, consistent with the polynorepinephrine structure, while the Au 4f region displayed characteristic doublets attributed to metallic Au⁰. These quantitative and high-resolution analyses provide direct evidence of the chemical composition and successful assembly of the hybrid interface.

### Electrochemical characterization

The tested materials underwent electrochemical characterization using the cyclic voltammetry (CV) technique, which enables the evaluation of redox properties. Measurements were performed in a potential range from.

**Fig. 4:**
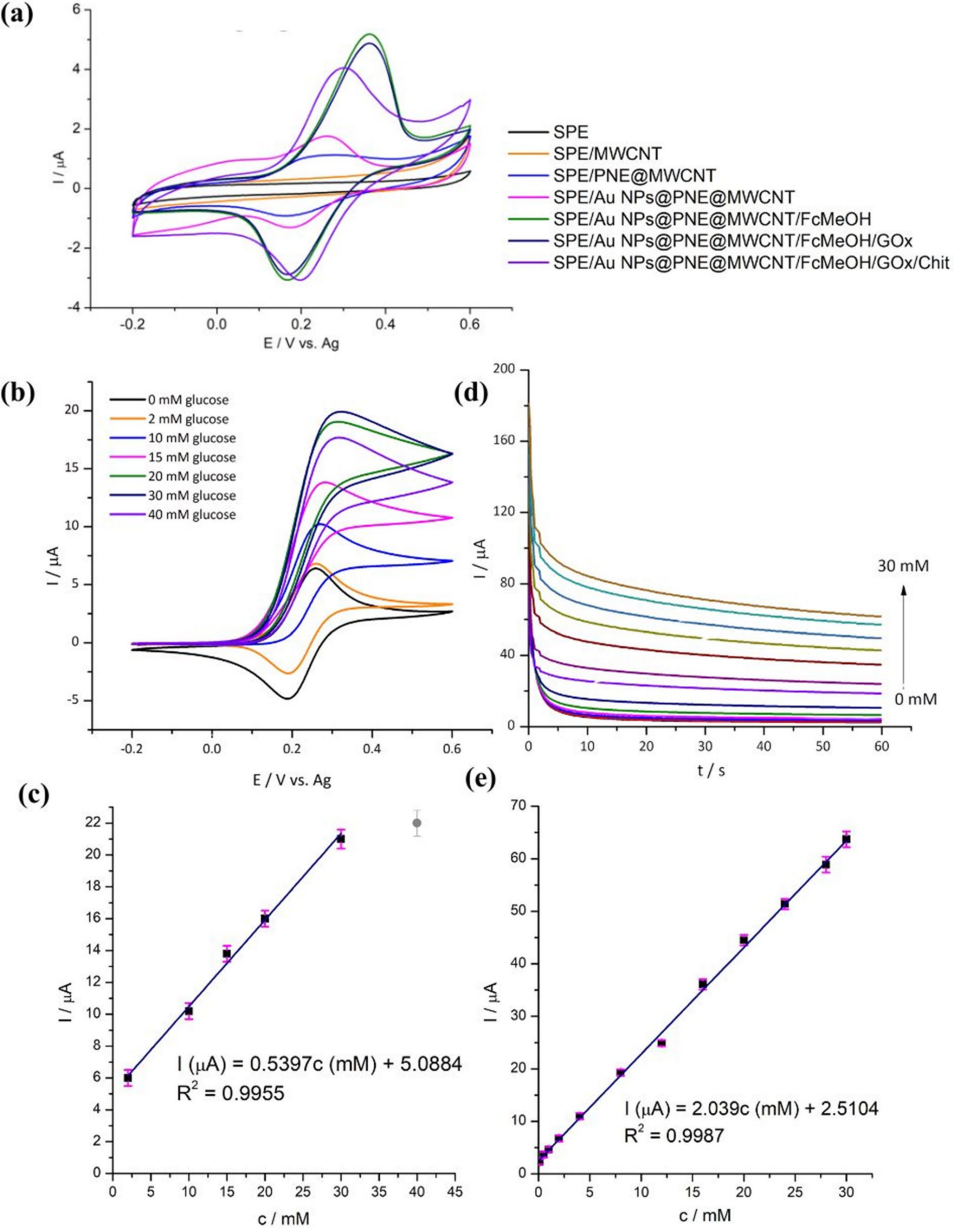
Electrochemical response for SPE electrode modification at individual stages (**a**) Au NPs@PNE@MWCNT/GOx with different glucose concentration (0–40 mM) (**b**); current dependence on concentration (**c**) (*n* = 3; SD = 0.84; SE = 0.38); chronoamperometric response of the hybrid material-based glucose biosensor to increasing glucose concentrations (0–30 mM) (**d**); calibration curve derived from steady-state current values (**e**) (*n* = 3; SD = 1.33; SE = 0.38) all graphs were recorded in PBS solution (50 mM; pH = 7.4)

–0.2 to 0.6 V relative to the reference electrode, at a scanning rate of 10 mV s⁻¹. The results are presented in Fig. 4a. For the bare electrode (SPE) and the electrode modified with MWCNT, no redox peaks were observed in the tested potential range. At the same time, an increase in current was observed after MWCNT modification, possibly indicating that the material has an increased active surface and improved capacitive properties, which is desirable in the context of further modification of the electrode with electrochemically active materials.

After modification of the MWCNT electrode with a polynorepinephrine layer, changes in the electrochemical characteristics can be observed in the CV graph, indicating the presence of active redox groups. Compared with the unmodified MWCNT electrode, well-defined anodic and cathodic peaks appear at 0.27 V and 0.15 V, respectively, which correspond to the oxidation and reduction processes of hydroxyl and quinone groups present in the polymer structure. The presence of these peaks indicates the quasi-reversible nature of the redox reactions occurring in the PNE layer (Fig. 4a).

Further modification of the PNE@MWCNT system with gold nanoparticles led to further changes in the electrochemical response of the electrode. A shift of the redox peaks to potentials of 0.26 V and 0.18 V was observed in the CV plot, which may be due to the influence of gold on the electronic environment of the redox groups and an improvement in the conductive properties of the system. The presence of Au NPs also promotes more efficient electron transfer, which was reflected in a significant increase in the peak currents for both the oxidation and reduction processes. The observed changes indicate a synergistic effect between the polymer layer and gold nanoparticles, which significantly improves the electrochemical activity of the electrode and makes it an even more attractive platform for applications in sensitive detection systems [34, 35].

In the next stage, under identical electrochemical conditions (CV, potential range − 0.2 to 0.6 V, scanning speed 10 mV s^−^¹), the individual stages of electrode modification were characterized. In the first step, the SPE-type electrode was modified with a hybrid material consisting of carbon nanotubes, polynorepinephrine, and gold nanoparticles (Au NPs@PNE@MWCNT), which served as a conductive and electroactive substrate for further functionalization stages. This compound was selected due to its good conductivity and chemical stability, the reversible Fc/Fc⁺ redox couple, and its relatively low redox potential (approximately 0.3 V), which enables operation under mild conditions and reduces non-specific oxidation reactions in biosensing systems [36–38].

After modification of (hydroxymethyl)ferrocene, a shift of the anodic peak potential to 0.32 V and a significant increase in peak currents were observed (Fig. 4a). The potential shift can be attributed to the presence of (hydroxymethyl)ferrocene as an additional, well-defined redox mediator, which has its own formal potential in the range of about 0.30–0.35 V.

In the next step, the enzyme glucose oxidase, responsible for specific recognition and oxidation of glucose, was applied to the electrode surface (Fig. 4a) [39]. The addition of GOx to the system resulted in a slight decrease in the current value, which can be attributed to the presence of a protein layer limiting access of mediators and analytes to the electrode surface and partially inhibiting electron transfer.

In order to stabilize the system and immobilize the enzyme, the system was covered with a thin layer of 1% chitosan solution. Chitosan serves here as a biocompatible matrix, which not only protects the enzyme from denaturation, but also ensures proper hydration of the enzyme layer and allows substrate diffusion [40]. The final coating of the electrode with 1% chitosan solution resulted in a further reduction in the current and a shift of the redox peak potentials to 0.20 V and 0.30 V, respectively.

To determine the mass transport mechanism in the studied electrochemical system, cyclic voltammetry measurements were performed at different scan rates ranging from 10 to 100 mV s⁻¹ in PBS (pH 7.4; 100 mM) in the presence of 3 mM glucose. The peak current data were converted to logarithmic form, and a plot of log(Ip) versus log(v) was constructed (Fig. S2; see Supplementary Material). Linear regression yielded a slope of 0.7555 (R² = 0.9963).

Since a slope of 0.5 corresponds to purely diffusion-controlled behavior and a value of 1.0 indicates adsorption-controlled kinetics, the obtained intermediate slope suggests a mixed adsorption–diffusion control. This indicates that, in addition to diffusion from the bulk solution, partial surface confinement or interfacial interactions of the electroactive species contribute to the overall electrochemical response [41]. This interpretation was further supported by the linear dependence of both oxidation and reduction peak currents on v^1/2^, confirming the contribution of diffusion-controlled transport (Fig. S2c).

Electrochemical impedance spectroscopy (EIS) was used to compare the interfacial properties of PNE@MWCNT/FcMeOH/GOx/Chit and Au NPs@PNE@MWCNT/FcMeOH/GOx/Chit in a 1 mM ferricyanide/ferrocyanide solution. The Nyquist plots (Fig. S3, see Supplementary Material) revealed similar solution resistance values (Rs ≈ 113 Ω) for both systems, indicating identical electrolyte conditions. However, electrode B exhibited a lower charge transfer resistance (Rct ≈ 24 Ω) compared to electrode A (Rct ≈ 28 Ω), suggesting enhanced electron transfer kinetics. The double-layer capacitance (Cdl) remained comparable (~ 4.5 × 10⁻⁵ F), indicating similar interfacial characteristics. The presence of a Warburg impedance in both cases confirms diffusion-controlled processes at low frequencies.

The improved performance of electrode with AuNPs can be attributed to the incorporation of gold nanoparticles, which are known to enhance electrical conductivity and facilitate electron transfer. The presence of AuNPs increases the effective electroactive surface area and provides additional conductive pathways, thereby reducing interfacial resistance. Moreover, gold nanoparticles can promote more efficient electron tunneling between the redox species and the electrode surface, as well as improve the organization of the sensing layer. These factors collectively contribute to the observed decrease in Rct and improved electrochemical performance [27, 39].

### Detection of glucose

The electrocatalytic response of the PNE@MWCNT/GOx and Au NPs@PNE@MWCNT/GOx modified electrodes to glucose oxidation was investigated using cyclic voltammetry. The glucose oxidation mechanism is based on the enzymatic reaction catalysed by glucose oxidase, in which glucose is oxidized to gluconolactone, while FAD is reduced to FADH₂.

The PNE@MWCNT/GOx-modified electrode exhibited a linear current response in the glucose concentration range 2–15 mM, while the Au NPs@PNE@MWCNT/GOx-modified electrode displayed an extended linear range from 2 to 30 mM. This indicates that the incorporation of Au nanoparticles significantly improved the sensor’s performance, enhancing its sensitivity and broadening the detection range, which is advantageous for practical glucose monitoring applications. The electrochemical results for glucose detection by the PNE@MWCNT/GOx system are presented in Fig. S4 (see Supplementary Material), while those for the Au NPs@PNE@MWCNT/GOx system are presented in Fig. 4c.

The superior linearity of the gold-containing composite can be attributed to the unique electrochemical properties of gold nanoparticles. Gold provides a highly conductive surface and facilitates enhanced electron transfer between the active sites of GOx and the electrode, reducing charge transfer resistance and promoting efficient redox cycling of the mediator. This makes Au NPs@PNE@MWCNT/GOx a more promising platform for biosensing applications that require high sensitivity and wide linearity.

Chronoamperometry was employed as an analytical technique to investigate the electrochemical response of the developed glucose biosensors. Measurements were performed by recording the current at a constant potential in the presence of an external redox mediator.

For the electrode modified with the Au NPs@PNE@MWCNT/GOx composite, an increase in current was observed over a glucose concentration range from 0.1 mM to 30 mM, with linear correlation (R² = 0.9987) (Fig. 4d). The calibration curve had a slope of 2.039 µA mM⁻¹, and the sensitivity was calculated to be.

28.86 µA mM^−^¹ cm^−^² (Fig. 4e). The limit of detection (LOD), calculated as three times the standard deviation of the blank signal divided by the slope, was determined to be 2.15 µM, and the limit of quantification (LOQ) was calculated as 6.52 µM, according to the equation LOQ = 3.3·(SD/a), where σ is the standard deviation of the blank signal and S is the slope of the calibration curve. The standard deviation (SD) for these measurements was 1.33, and the standard error (SE) was 0.38. These results reflect a highly sensitive and stable biosensor with excellent electrocatalytic performance over an extended working range.

Pearson’s correlation analysis was performed to quantify the strength of the relationship between glucose concentration (independent variable) and the measured electrochemical current response (dependent variable), while the reported standard deviations were used only to describe measurement precision and were not included in the calculation. The analysis yielded a Pearson correlation coefficient of *r* = 0.998, indicating an extremely strong positive association between the variables.

For comparison, the electrode modified with PNE@MWCNT/GOx (without gold nanoparticles) demonstrated linearity in a narrower concentration range, from 1 mM to 20 mM, with a slightly lower correlation coefficient (R² = 0.9918). The chronoamperometric curves are shown in Fig. S5a (see Supplementary Material). The slope of the calibration curve was 0.7875 µA mM^−^¹, resulting in a calculated sensitivity of.

11.15 µA mM^−^¹ cm^−^². The LOD for this sensor was calculated as 4.15 µM, and the LOQ as 12.58 µM under the same assumptions. The graph showing the current dependence of glucose concentration on the nanomaterial without gold nanoparticles is presented in Fig. S5b (see Supplementary Material).

The extension of the linear detection range to 0.1–30.0 mM achieved by AuNP incorporation is particularly relevant from a clinical perspective. This range fully covers physiological, postprandial, and pathological glucose concentrations encountered in routine diabetes diagnostics and monitoring. Notably, the increased upper linearity limit prevents signal saturation at elevated glucose levels (> 20 mM), which is critical for accurate assessment in cases of severe hyperglycemia.

Although both PNE@MWCNT- and Au NPs@PNE@MWCNT-based biosensors exhibit effective glucose sensing, the incorporation of gold nanoparticles results in improved analytical performance. The AuNP-modified system shows higher sensitivity and a wider linear range within the clinically relevant concentration window.

The proposed glucose biosensor demonstrates competitive analytical performance compared with solutions described previously in the literature in terms of sensitivity, linearity range, and limit of quantification. A detailed comparison of selected analytical parameters is presented in Table 1.

**Table 1:** Comparison of the proposed lab biosensor with other GOx-based systems from the literature

Electrode	Electrochemical technique	Linear range (mM)	Limit of detection (µM)	Sensitivity (µA/mM cm^− 2^)		Practical applicability	Ref.
GC/Fe_3_O_4_@PDA-GOx	Amperometry	1.0–20.0	1.54	139.71		Model glucose solutions	[36]
GOx-gel-rGO-Au/SPGE	Amperometry	0.85–7.72	1.25	53.70		Human sweat analysis, wearable sensing	[42]
PGOx@MXene/CS	Amperometry	0.03–16.5	3.10	48.98		Whole blood analysis	[43]
PANI-MWCNT/PPy/GOx	Amperometry	0.01–2.0	2.38	81.74	Blood serum samples		[44]
GOx/NPG/GCE	DPV	0.05–10.0	1.02	12.10	Serum sample		[45]
GR/DGNS/Cys/GOx	Amperometry	0.05–1.0	27.0	93.70	Serum sample		[46]
MWCNT/Nafion/GOx/GCE	Amperometry	1.0–3.0	4.94	32.40	Honey sample analysis		[47]
GC/MWCNT/Fe_3_O_4_/PDA-GOx	Amperometry	2.0–20.0	2.25	5.04	Long-term glucose monitoring		[48]
PNE@MWCNT/GOx	Amperometry	1.0–20.0	4.15	11.15	Model glucose solutions		This work
Au NPs@PNE@MWCNT/GOx	Amperometry	0.1–30.0	2.15	28.86	Whole blood analysis, wireless POCT monitoring, intra-day glucose tracking		This work

The improved analytical performance of the Au-containing electrode can be attributed to the incorporation of gold nanoparticles, which improve electrical conductivity and facilitate efficient electron transfer between FADH₂ and the electrode surface. Moreover, the gold nanoparticles likely improve enzyme immobilization and orientation, further promoting biocatalytic activity and signal stability.

### Interference study

In glucose biosensor studies, it is essential to assess the influence of potential interferents, such as ascorbic acid and uric acid, which may cause electrochemical signals and disrupt the selectivity of detection.

**Fig. 5:**
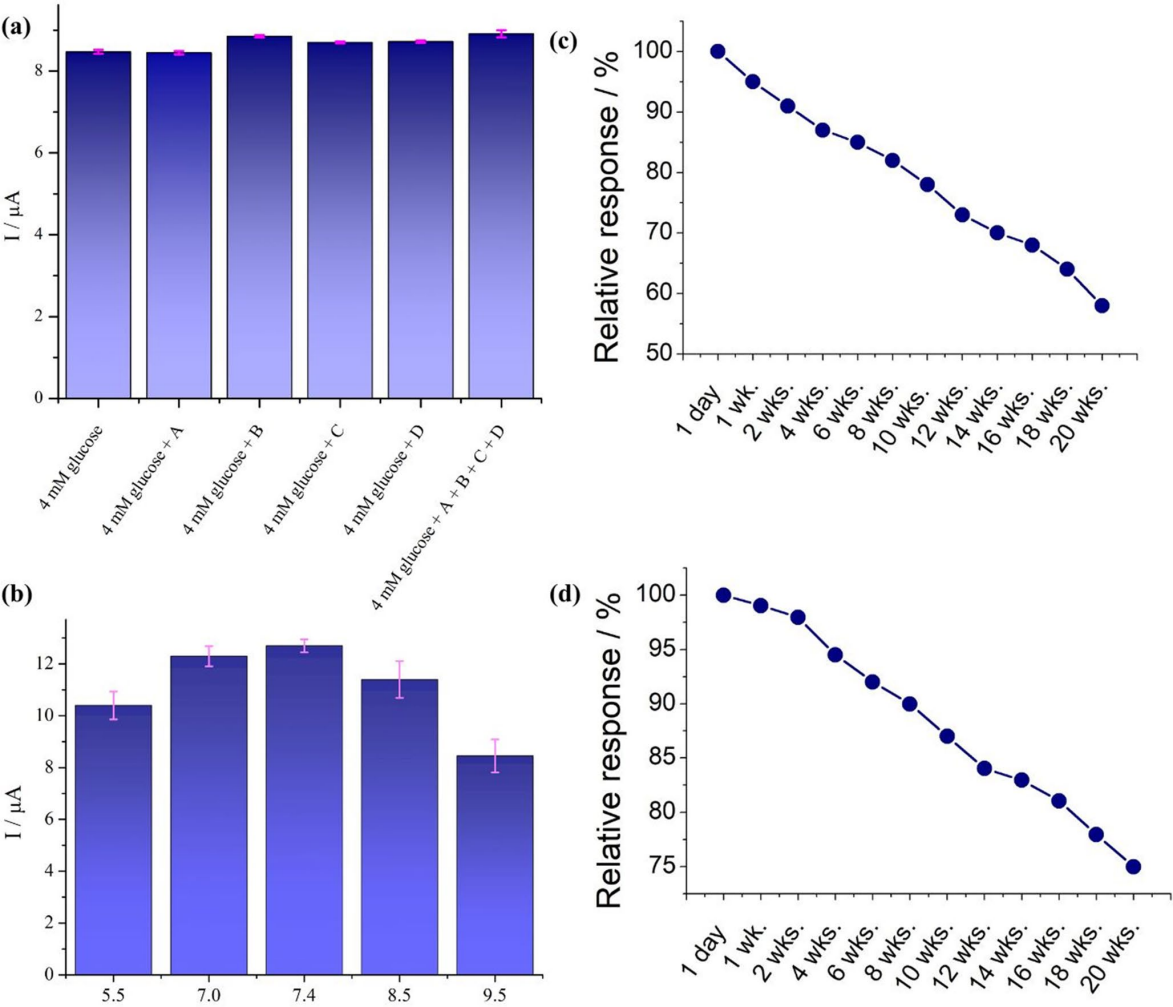
Chronoamperometric response for 4 mM glucose and the following interferents: 4 mM fructose (A), 0.1 mM ascorbic acid (B), 0.2 mM uric acid (C), 0.2 mM dopamine (D), and a mixture of glucose and all interferents (A + B + C + D) (*n* = 3) (**a**); influence of various pH (5.5; 7.0; 7.4; 8.5; 9.5) in the presence of 5 mM glucose (*n* = 3) (**b**); graph showing the change in relative biosensor response up to 20 weeks without (**c**), and with Au NPs (**d**) for 3 mM of glucose recorded in PBS solution (50 mM; pH = 7.4)

The effect of potential interfering agents, including fructose, ascorbic acid, dopamine, and uric acid, was evaluated by chronoamperometry. Tests were performed to compare the biosensor response to glucose with that recorded in the presence of glucose and a single interferent, as well as a mixture of all tested interferents. The results showing the individual responses are shown in Figs. S6–S11 (see Supplementary Material), and collective results are shown in Fig. 5a.

The effect of fructose was studied to assess the selectivity of the biosensor towards glucose, given the structural similarity of these two sugars and the possibility of co-occurrence in biological samples. As can be seen in Fig. 5a, fructose produced no changes in the electrochemical response, confirming the selectivity of the enzyme against other sugars.

Ascorbic acid, dopamine, and uric acid were chosen as interferents because of their electroactivity and common occurrence in body fluids such as blood and saliva, making them significant interfering factors in the electrochemical determination of glucose. To evaluate these compounds’ current response, measurements were performed in solutions containing glucose (4 mM) along with typical interferents found in biological matrices: dopamine (0.1 mM), ascorbic acid (0.1 mM), uric acid (0.2 mM), and a mixture of all of these compounds. In all cases, the glucose concentration was maintained at 4 mM to assess the influence of each interferent on the sensor’s ability to detect glucose. The recorded current changes (ΔI) were as follows: 8.47 µA for glucose, 8.48 µA for glucose with fructose, 8.72 µA for glucose with dopamine, 8.85 µA for glucose with ascorbic acid, 8.70 µA for glucose with uric acid, and 8.90 µA for the mixture containing glucose and all interferents. In the presence of mixed interfering species, only a slight increase in the current response was observed (8.90 µA compared to 8.47 µA for glucose alone), corresponding to an approximately 5% deviation. Considering the complexity of multicomponent biological environments, this variation indicates satisfactory selectivity and does not significantly affect the analytical performance of the developed biosensor [49, 50]. These results indicate that the biosensor exhibits good selectivity and robustness against interference from common electroactive species.

### Effect of pH on Biosensor Performance

The performance of the glucose biosensor was evaluated at different pH values (5.5, 7.0, 7.4, 8.5, and 9.5) in the presence of 5 mM glucose (*n* = 3), the results are presented in Fig. 5b. The highest sensor response was observed at pH 7.4, indicating that near-physiological conditions are optimal for the enzymatic and electrochemical processes involved. At more acidic and alkaline pH values, a decrease in signal was noted, which may be attributed to reduced enzyme activity and altered interfacial properties of the sensing layer [51, 52].

### Time stability

The time stability of glucose biosensors remains a significant challenge for glucometers and other biosensing systems. Over time, sensors may lose sensitivity and accuracy due to the degradation of enzymes and structural changes in the electrode materials. Moreover, environmental factors such as temperature and humidity can further accelerate the aging process of the sensors [48, 53].

The stability of the developed glucose biosensor was assessed based on the changes in the electrochemical signal (∆I) in response to a constant glucose concentration (3 mM) measured once a week for a period of 20 weeks (Fig. S11; see Supplementary Material).

The initial current response of the biosensor on the first day was 8.39 µA, which was defined as a 100% relative response. After four weeks, the current intensity had decreased slightly to 7.94 µA, which was 98% of the initial response, indicating high performance stability in the first month of use.

After 20 weeks, the biosensor maintained 75% of the initial response, which still indicates good long-term stability. For comparison, according to FDA guidelines and literature data for commercial glucometers, devices approved for home use should maintain measurement reliability within ± 15% for 95% of results over the declared shelf life, typically ranging from 3 to 6 months. Therefore, maintaining 75% of the initial response after five months suggests promising storage stability for POCT-oriented applications, although further optimization and periodic recalibration may still be required for long-term clinical use and patient self-monitoring applications [54–56].

For comparative purposes, a stability test over time was also performed for the system without the addition of gold nanoparticles. The relative response for this system is shown in Fig. 5c. Similarly, these tests were performed using chronoamperometry, at a constant potential and with the addition of 3 mM glucose, and then ΔI was read for the systems (Fig. S13; see Supplementary Material). The system produced a result below 75% after 12 weeks (73%), and after 20 weeks the relative response of the system was 58%.

Figure 5c and d illustrate the evolution of the relative biosensor response over 20 weeks, expressed as a percentage of the initial signal. The comparison clearly demonstrates enhanced long-term stability of the AuNP-decorated system, which retains ~ 75% of its initial response after 20 weeks, compared to ~ 58% for the AuNP-free sensor. The use of gold nanoparticles in the MWCNT@PNE composite significantly improved the biosensor’s time-stability compared with a system without gold. The presence of Au NPs promoted structural stabilization of the active layer for immobilized biomolecules, limiting their desorption and degradation during storage and operation of the biosensor [57]. As a biocompatible material, gold also acted as an environmental stabilizer for the biomolecules, limiting their denaturation and loss of biological activity [46]. The synergistic combination of Au NPs with the MWCNT@PNE matrix contributed to a significant extension of the biosensor’s functional lifetime and improved its stability under long-term conditions.

The Au NPs-doped system exhibits clear advantages over the Au NPs-free platform, providing several cumulative analytical benefits. Compared to the undoped configuration, the Au NPs@PNE@MWCNT-based biosensor demonstrates a (i) broader linear detection range (0.1–30.0 mM vs. 1–20 mM), (ii) more than a two-fold increase in sensitivity (28.85 vs. 11.15 µA mM^−^¹ cm^−^²), (iii) a lower limit of detection, and (iv) markedly improved long-term stability, making it a more efficient and reliable analytical platform.

### Repeatability and reproducibility

**Fig. 6:**
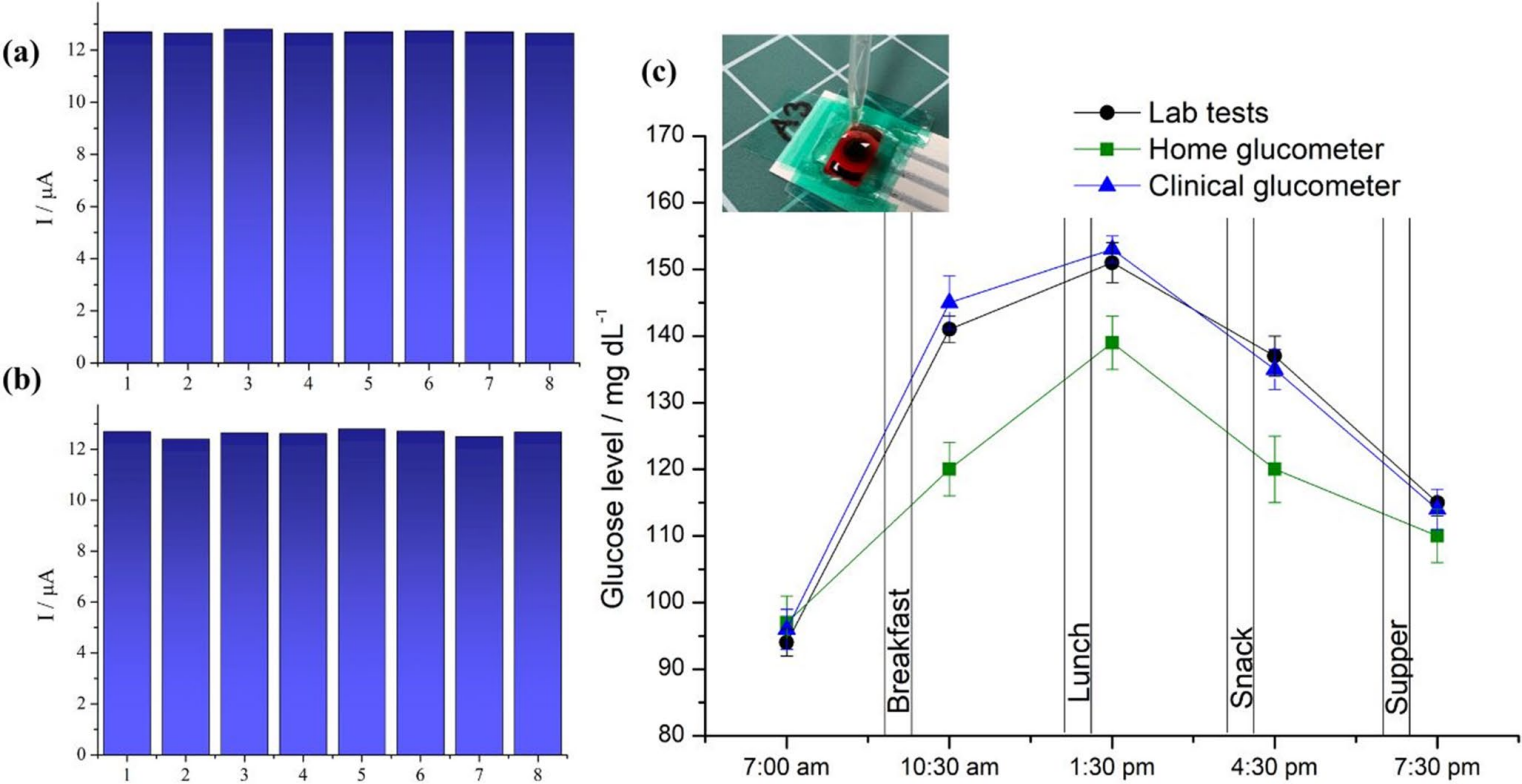
Repeatability (**a**) and reproducibility (**b**) of the glucose biosensor based on chronoamperometric measurements; comparison of human blood glucose level measurements throughout the day in the lab tests vs. results from the home glucometer and clinical glucometer (**c**) (*n* = 3) attached: photo of the measurement with the electrode shown recorded in PBS solution (100 mM; pH = 7.4)

The repeatability and reproducibility of the biosensor system were evaluated using chronoamperometry based on eight consecutive measurements, performed in PBS buffer (pH 7.4; 100 mM) in the presence of 5 mM glucose. The biosensor system demonstrated excellent repeatability with a mean response of 12.70 ± 0.05 (SD, *n* = 8). The relative standard deviation (RSD) was 0.41%, confirming high measurement precision. The standard error of the mean (SE) was 0.018. The results are presented in Fig. 6a.

The biosensor system showed good reproducibility with a mean response of 12.63 ± 0.13 (SD, *n* = 8). The relative standard deviation (RSD) was 1.01%, indicating high measurement precision. The standard error of the mean (SE) was 0.045. All measurements were performed in the presence of 5 mM glucose, and the results are presented in Fig. 6b.

### Real samples

Glucose detection in real samples is essential for evaluating sensor performance under practical conditions. Due to the complex matrix of biological fluids, which is subject to such factors as potential interferents and varying ionic strength, it is essential for a biosensor to achieve high selectivity and stability.

In this study, chronoamperometric measurements were used to assess the sensor’s response in human serum samples, demonstrating its ability to offer reliable glucose quantification in complex biological environments. Fresh capillary blood samples were analyzed immediately after collection, and the small sample volume together with the short measurement time minimized the influence of blood cells during electrochemical analysis. The results are presented in Table 2.

**Table 2:** Biosensor response in human serum and comparison with commercial glucometer results (*n* = 3)

Glucose concentration / mg dL^− 1^	Clinical glucometer / mg dL^− 1^	Home glucometer / mg dL^− 1^	Lab tests / mg dL^− 1^
80	78 ± 3	72 ± 6	78 ± 1
110	100 ± 2	122 ± 4	102 ± 3
120	119 ± 3	134 ± 5	116 ± 4
130	123 ± 2	113 ± 5	125 ± 2
140	131 ± 4	126 ± 3	129 ± 4

In the next stage of the study, real measurements were conducted by analysing glucose levels in human blood using three instruments: the proposed laboratory biosensor, a home glucometer, and a clinical glucometer. The first measurement was performed in a fasting state, where the lab biosensor indicated a value of 94 mg dL^− 1^. The subsequent measurement was taken 30 min after breakfast (an apple and a cup of coffee), yielding a glucose concentration of 141 mg dL^− 1^. After a meal consisting of wheat pasta and vegetables, glucose increased up to 151 mg dL^− 1^. Following a snack consisting of a banana and a cup of tea, the glucose level was recorded at 137 mg dL^−^¹, and after the final meal, which was a vegetable salad, the level decreased to 115 mg dL^−^¹.

These results demonstrate that the proposed biosensor reliably captured the increase in glucose concentration in human blood following food intake and the gradual decline between meals. The sensor’s response was consistent with the physiological profile of postprandial glycemia in a healthy individual without diabetes. Glucose concentration increased after breakfast, reaching its highest value around lunchtime (approximately 150–160 mg dL^−^¹ for lab and clinical measurements). Afterward, the glucose level gradually declined following the afternoon snack and supper.

In Fig. 6c, the results obtained with the laboratory biosensor are presented and compared with those of the home and clinical glucometers.

Comparison of the three measurement approaches revealed that values obtained with the laboratory biosensor closely matched those of the clinical glucometer, confirming the high reliability of the developed solution. In contrast, the home glucometer showed slight deviations from the clinical measurements, although it maintained a comparable trend in glucose dynamics. It should be noted that clinical glucometers provide higher sensitivity and accuracy due to advanced calibration procedures, stringent quality control, and stable measurement conditions [58]. Home glucometers, while practical and fast, are more prone to variability arising from simplified technology and potential user errors [59].

The study confirms that the laboratory biosensor effectively monitors blood glucose fluctuations in response to dietary intake under non-diabetic conditions. Its performance is comparable to that of a clinical glucometer, highlighting its potential applicability in diagnostic and metabolic monitoring contexts.

## Conclusions

In this study, a novel enzymatic glucose biosensor based on an AuNPs@PNE@MWCNT/GOx hybrid nanocomposite was successfully developed for point-of-care glucose monitoring. The proposed platform combines the high conductivity of AuNPs and MWCNTs with the favorable enzyme immobilization properties of polynorepinephrine, resulting in enhanced electrochemical performance and sensing stability. The fabricated biosensor exhibited good sensitivity, selectivity, a wide linear range, and satisfactory long-term stability, retaining approximately 75% of the initial response after 20 weeks. Moreover, successful measurements in real blood samples and wireless real-time signal acquisition confirmed its practical applicability for POCT applications. Compared with previously reported catecholamine-based biosensors, the developed platform offers the combined advantages of efficient electron transfer, prolonged stability, and practical usability. Although the system remains dependent on enzyme stability, the proposed sensing strategy provides a promising platform for future portable biosensors and may be extended toward the detection of other oxidoreductase-related analytes, such as cholesterol or lactate.

## Supplementary Information

Below is the link to the electronic supplementary material.

Supplementary Material 1

## Data Availability

No datasets were generated or analysed during the current study.
