# Supplementary material for: Bioinspired catecholamine-derived AuNPs@polynorepinephrine@MWCNT electroactive nanomaterial for real-time monitoring of glucose dynamics
Source: Mikrochim Acta. 2026 Jun 17;193(7):479. doi: 10.1007/s00604-026-08201-5 (PMC13275580; doi:10.1007/s00604-026-08201-5)
Supplement: Supplementary file 1 — Supplementary Material 1 [file 604_2026_8201_MOESM1_ESM.docx]

**Supplementary material**

**Bioinspired catecholamine-derived AuNPs@polynorepinephrine@MWCNT electroactive nanomaterial for real-time monitoring of glucose dynamics**

Artur Jędrzak^1,*^, Maria Kuznowicz^2^, Teofil Jesionowski^1^

^1^Institute of Chemical Technology and Engineering, Faculty of Chemical Technology,

Poznan University of Technology, Berdychowo 4, PL–60965 Poznan, Poland

^2^Interdisciplinary Centre for Ecotechnology, Poznan University of Technology,
PL–60965 Poznan, Poland

*Corresponding author: artur.jedrzak@put.poznan.pl

**Table S1.** Comparison of commercially used blood glucose meters

| Parameter | iXell | Wellion CALLA Light |
| --- | --- | --- |
| Assay type | Enzymatic electrochemical assay (glucose oxidase–based biosensor, whole capillary blood) | Enzymatic electrochemical assay (glucose oxidase–based biosensor, whole capillary blood) |
| Intended use / positioning | Clinical / professional and point-of-care use, as well as supervised home monitoring | Primarily home-use / self-monitoring blood glucose system |
| Analysis time | ~7 seconds | ~6 seconds |
| Sample volume | ~0.7 µL | ~0.65 µL |
| Measurement range | 20-600 mg/dL (1.1–33.3 mmol/L) | 20-600 mg/dL (1.1–33.3 mmol/L) |


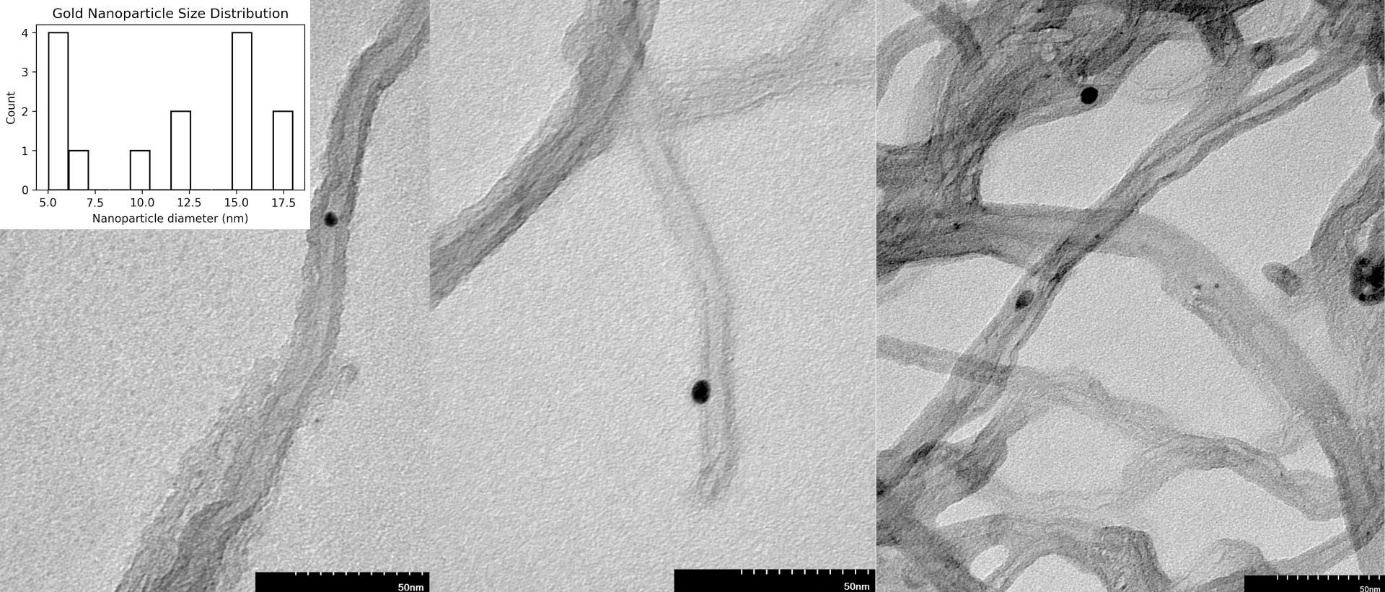


**Fig. S1.** TEM image of Au NPs@PNE@MWCNT with size distribution histogram





**Fig. S2.** Cyclic voltammograms of SPE/Au NPs@PNE@MWCNT/GOx in PBS (pH 7,4; 100 mM) in the presence of 3 mM glucose at different scan rates (in the range from 10 to 100 mV s^−1^) (a); linear fitting chart of log scan rate and log I (b); presents the corresponding I vs. v^1/2^ relationship (c) (n=3)





**Fig. S3.** Nyquist plots of PNE@MWCNT/FcMeOH/GOx/Chit and AuNPs@PNE@MWCNT/FcMeOH/GOx/Chit electrodes recorded in 1 mM ferricyanide/ferrocyanide solution.





**Fig. S4.** Cyclic voltammetry against increasing glucose concentrations for the PNE@MWCNT/GOx biosensor without gold (a); calibration curve for the presented system (b) (n=3; SD = 0.25; SE = 0.13)





**Fig. S5.** Chronoamperometric response of the glucose biosensor based on the gold-free material to various glucose concentrations (a); corresponding calibration curve obtained from steady-state current values (b) (n=3; SD=0.99; SE = 0.35)





**Fig. S6.** Chronoamperometric response of the proposed biosensor for 4 mM glucose


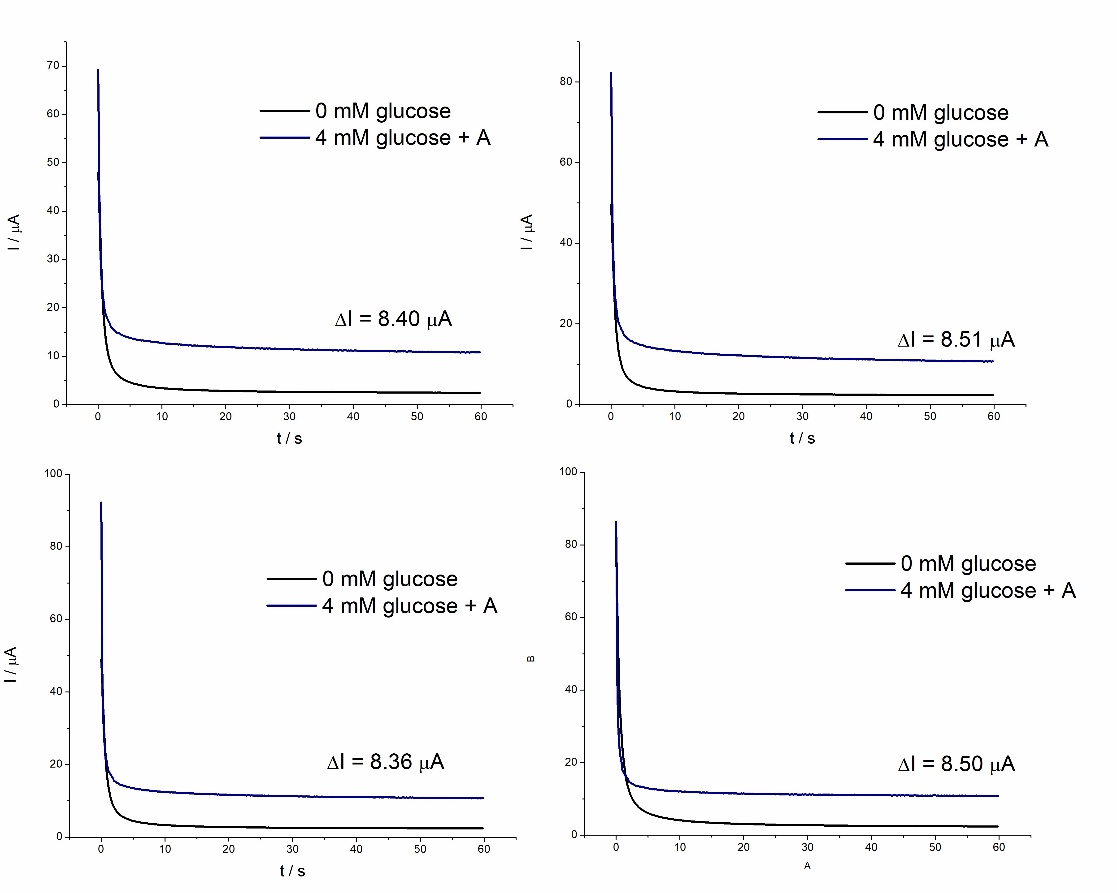


**Fig. S7.** Chronoamperometric response of the proposed biosensor for a mixture containing 4 mM glucose
and 4 mM fructose (A)





**Fig. S8.** Chronoamperometric response of the proposed biosensor for a mixture containing 4 mM glucose
and 0.1 mM ascorbic acid (B)





**Fig. S9.** Chronoamperometric response of the proposed biosensor for a mixture containing 4 mM glucose
and 0.2 mM uric acid (C)





**Fig. S10.** Chronoamperometric response of the proposed biosensor for a mixture containing 4 mM glucose
and 0.2 mM dopamine (D)





**Fig. S11.** Chronoamperometric response of the proposed biosensor for a mixture containing 4 mM glucose, 4 mM fructose (A), 0.1 mM ascorbic acid (B), 0.2 mM uric acid (C), and 0.2 mM dopamine (D)








**Fig. S12.** Chronoamperometric plot for the response of Au NPs@PNE@MWCNT/GOx system to 3 mM glucose after 1 day (a); 1 week (b); 2 weeks (c); 4 weeks (d); 6 weeks (e); 8 weeks (f); 10 weeks (g); 12 weeks (h); 14 weeks (i); 16 weeks (j); 18 weeks (k); 20 weeks (l)








**Fig. S13.** Chronoamperometric curves for the response of PNE@MWCNT/GOx system to 3 mM glucose after 1 day (a); 1 week (b); 2 weeks (c); 4 weeks (d); 6 weeks (e); 8 weeks (f); 10 weeks (g); 12 weeks (h); 14 weeks (i); 16 weeks (j); 18 weeks (k); 20 weeks (l)
